# Geospatial investigations in Colombia reveal variations in the distribution of mood and psychotic disorders

**DOI:** 10.1038/s43856-024-00441-x

**Published:** 2024-02-21

**Authors:** Janet Song, Mauricio Castaño Ramírez, Justin T. Okano, Susan K. Service, Juan de la Hoz, Ana M. Díaz-Zuluaga, Cristian Vargas Upegui, Cristian Gallago, Alejandro Arias, Alexandra Valderrama Sánchez, Terri Teshiba, Chiara Sabatti, Ruben C. Gur, Carrie E. Bearden, Javier I. Escobar, Victor I. Reus, Carlos Lopez Jaramillo, Nelson B. Freimer, Loes M. Olde Loohuis, Sally Blower

**Affiliations:** 1 Center for Neurobehavioral Genetics, Semel Institute for Neuroscience and Human Behavior, David Geffen School of Medicine, University of California Los Angeles, Los Angeles, CA USA; 2 Department of Mental Health and Human Behavior, University of Caldas, Manizales, Colombia; 3 Center for Biomedical Modeling, Semel Institute for Neuroscience and Human Behavior, David Geffen School of Medicine, University of California, Los Angeles, CA USA; 4 Department of Psychiatry, University of Antioquía, Medellín, Colombia; 5 Department of Biomedical Data Science, Stanford University, Stanford, CA USA; 6 Department of Psychiatry, University of Pennsylvania School of Medicine and the Penn-CHOP Lifespan Brain Institute, Philadelphia, PA USA; 7 Department of Psychiatry, Herbert Wertheim College of Medicine, Florida International University, Miami, FL USA; 8 Department of Psychiatry, University of California San Francisco, San Francisco, CA USA

**Keywords:** Health services, Computational neuroscience, Computational neuroscience

## Abstract

**Background:**

Geographical variations in mood and psychotic disorders have been found in upper-income countries. We looked for geographic variation in these disorders in Colombia, a middle-income country. We analyzed electronic health records from the Clínica San Juan de Dios Manizales (CSJDM), which provides comprehensive mental healthcare for the one million inhabitants of Caldas.

**Methods:**

We constructed a friction surface map of Caldas and used it to calculate the travel-time to the CSJDM for 16,295 patients who had received an initial diagnosis of mood or psychotic disorder. Using a zero-inflated negative binomial regression model, we determined the relationship between travel-time and incidence, stratified by disease severity. We employed spatial scan statistics to look for patient clusters.

**Results:**

We show that travel-times (for driving) to the CSJDM are less than 1 h for ~50% of the population and more than 4 h for ~10%. We find a distance-decay relationship for outpatients, but not for inpatients: for every hour increase in travel-time, the number of expected outpatient cases decreases by 20% (RR = 0.80, 95% confidence interval [0.71, 0.89], *p* = 5.67E-05). We find nine clusters/hotspots of inpatients.

**Conclusions:**

Our results reveal inequities in access to healthcare: many individuals requiring only outpatient treatment may live too far from the CSJDM to access healthcare. Targeting of resources to comprehensively identify severely ill individuals living in the observed hotspots could further address treatment inequities and enable investigations to determine factors generating these hotspots.

## Plain language summary

The frequencies of mental disorders vary by geographic region. Investigating such variations may lead to more equitable access to mental healthcare and to scientific discoveries that reveal specific localized factors that contribute to the causes of mental illness. This study examined the frequency of three disorders with a major impact on public health – schizophrenia, bipolar disorder, and major depressive disorder – by analyzing electronic health records from a hospital providing comprehensive mental health care for a large region in Colombia. We show that individuals receiving outpatient care mainly live relatively near the facility. Those receiving inpatient care live throughout the region, but cluster in a few scattered locations. Future research could lead to strategies for more equitable provision of mental healthcare in Colombia and identify environmental or genetic factors that affect the likelihood that someone will develop one of these disorders.

## Abstract (web summary)

Song et al. conduct a geospatial analysis of variations in incidence of mood and psychotic disorders and access to care in Caldas, Colombia. They show that many patients requiring only outpatient care live too far away to access it and identify hotspots of more severely ill patients, highlighting the need for targeted interventions.

## Introduction

Geographical variation in the distribution of mood and psychotic disorders has been observed, and extensively studied, in upper-income countries (UICs)^1–5^. These observed patterns may reflect inequities in the geographic accessibility of mental healthcare. Such inequities were first observed, and quantified, in a study published in 1852^1^. This, and subsequent studies^3,5–7^ used statistical models to identify a relationship between mental healthcare utilization and distance. Specifically, these studies identified the phenomenon termed distance-decay, whereby the utilization of a healthcare facility declines in direct proportion to the distance (or travel-time^8^) to the facility^1,2^. Studies in UICs have also indicated that geographical variations in the observed frequency of particular mental health disorders and outcomes may reflect localized social, environmental, and genetic factors associated with treatment utilization or disease risk^9–15^.

The longstanding availability in UICs of population registries and other public databases has provided crucial resources that have enabled geospatial investigation of treatment for mental health disorders, as exemplified by the several studies referenced above. A lack of comparable data repositories has traditionally limited the opportunities for conducting such research in low- and middle-income countries (LMICs). Recently, however, electronic health record (EHR) databases have become available in some LMICs and enable population-level investigations of individuals who have sought treatment for these disorders. As we describe here for a middle-income country, Colombia, analyses of such databases may provide insights that could not be obtained using previously available forms of data.

The main sources of population-level data on mental health disorders in Colombia have been four National Mental Health Surveys, conducted, most recently, in 2015^16,17^. The 2015 National Survey is an observational cross-sectional household-survey conducted in a nationally representative sample of 15,351 individuals aged seven and above, which obtained interview and self-report data. It has provided information on a wide range of topics (e.g., the prevalence of mental health and substance use disorders and on perceptions of access to care) that are important for our understanding of mental health disorders in Colombia and for efforts to improve the provision of mental healthcare.

Several critical types of information, however, are not available from the National Survey, but may be obtained by analyzing EHR databases; these include the distribution of the most severe and acute presentations of mental health disorders (the National Survey obtained little data on psychotic disorders), and the actual utilization of mental healthcare services. Additionally, as the National Survey sampled sparsely in any given location, its data cannot be used for geospatial investigation of mental health disorders. In contrast, as we demonstrate here, analyses utilizing the EHR database of the Clínica San Juan de Dios Manizales (CSJDM), provide a detailed picture of geospatial variation in treated mental illness across the entire department (state) of Caldas.

The CSJDM provides comprehensive mental healthcare for the one million inhabitants of Caldas. Located in the metropolitan municipality of Manizales, it provides mental health services to all individuals seeking care, regardless of health insurance status or other socioeconomic factors. We have identified and characterized geographic variation in the number of individuals who have sought treatment for the mood and psychotic disorders that most commonly result in psychiatric hospitalizations (bipolar disorder [BPD], schizophrenia [SCZ], and major depressive disorder [MDD]) and quantified differences, over the department, in geographic accessibility to mental healthcare, considering both individuals whose illness required inpatient care as well as those who were treated only as outpatients. We did so by combining diagnostic code data from the CSJDM EHR for the years 2005–2018, with publicly available geospatial data.

We show that travel-times (for driving) to the CSJDM are less than 1 h for ~50% of the population and more than 4 h for ~10%. We find a distance-decay relationship for outpatients, but not for inpatients: for every hour increase in travel-time, the number of expected outpatient cases decreases by 20%, an effect that is primarily driven by outpatients with MDD. Further, we identify nine hotspots where inpatient cases cluster. Notably, one hotspot comprises a nearly six-fold overrepresentation of inpatient BPD cases and crosses the administrative boundaries of two municipalities.

The results of the geospatial analyses that we performed can inform the design of programs aimed at reducing inequities in access to mental healthcare for mood and psychotic disorders. Additionally, they suggest the need for future studies aimed at identifying mechanisms for the geographical variations in disease incidence that we observed across the department of Caldas.

## Methods

### Inclusion criteria

We employed a retrospective cross-sectional design. Since 2005 the CSJDM has maintained EHRs on all outpatient visits and inpatient admissions. We utilized three forms of structured EHR data covering 2005–2018: demographic information, residential address, and diagnostic codes using the International Statistical Classification of Diseases and Related Health Problems: 10th Revision (ICD-10)^18^. The Institutional Review Boards (IRBs) at CSJDM and UCLA provided approval for the study, which involved uploading a version of the EHR database with patient names, dates (of birth and healthcare service), and with ID numbers removed, to a HIPAA-compliant Amazon Web Services server. Individual informed consent was not required for this study since only anonymized data were used. Gender was obtained through self-report and used as recorded in the EHR.

Using the EHRs for all adult patients 18–90 years, residing anywhere in Caldas, we focused on the 16,376 individuals who were assigned a diagnosis, at their first visit of BPD (diagnostic code: F31), SCZ (diagnostic code: F20), or MDD (diagnostic codes: F32 or F33). We used the initial diagnoses to eliminate possible bias introduced by right censoring of the diagnostic data, and did not use the longitudinal, repeated measures aspect of the EHR data in these analyses. In prior work, we have shown that diagnostic codes recorded in this EHR are highly accurate compared to diagnoses obtained by manual chart review by a psychiatrist^19^. We stratified analyses based on hospitalization status.

### Geocoding workflow

We identified the subset of diagnosed individuals with available residential addresses (*N* = 16,308). We assigned longitude and latitude coordinates to each patient’s address using the Spanish language setting of OpenCage’s geocoder through the R package opencage^20^. As input, OpenCage takes an address as free text. Prior to geocoding, we formatted addresses according to the following procedures. Most addresses (*N* = 11,745) fit within a grid pattern that is typical throughout Colombia, comprised of parallel streets called “calles” and perpendicular streets called “carreras”. We formatted addresses of this type according to the pattern “Calle [X], Carrera [Y], [Municipality], Caldas”, where X is the street number for the “calle”, and Y is the street number for the “carrera”. The remaining addresses (*N* = 4563) were situated in small rural areas called “veredas” which have few roads. For these addresses, we took the name of the vereda or other address entry and appended the name of the municipality and department.

OpenCage provides a list of potential longitude and latitude coordinates with corresponding accuracy confidence scores. If multiple potential matches were returned, the most relevant was selected, based on the OpenStreetMap data^21^ relevance criteria for Colombian addresses. We excluded addresses with OpenCage confidence scores lower than 6. Using this threshold for confidence scores ensured that all addresses included in the study were within a 7.5 km bounding box measured diagonally and yielded geographic coordinates for 16,295 of the 16,308 patient addresses; all analyses reported here focus on these 16,295 patients.

Table 1 provides a summary of the dataset stratified by diagnosis (BPD, SCZ, or MDD), gender, and hospitalization status. All individuals had the location of their residence georeferenced to a 7.5 × 7.5 km bounding box; for 71% of them we could define the location of their residence to a 0.5 × 0.5 km bounding box.

**Table 1:** Summary of the dataset.

	Inpatients (*n* = 5218)		Outpatients (*n* = 11,077)		All Patients (*n* = 16,295)	
	Men	Women	Men	Women	Men	Women
Total	2225 (43%)	2993 (57%)	3512 (32%)	7565 (68%)	5737 (35%)	10558 (65%)
BPD	861 (38%)	1431 (62%)	937 (35%)	1756 (65%)	1798 (36%)	3187 (64%)
MDD	979 (40%)	1476 (60%)	2157 (28%)	5645 (72%)	3136 (31%)	7121 (69%)
SCZ	385 (82%)	86 (18%)	418 (72%)	164 (28%)	803 (76%)	250 (24%)

Total number of patients included in study by diagnosis (BPD, MDD, and SCZ), severity (inpatient vs outpatient), and gender. Only individuals with complete information were included in the study.

### Calculating the incidence rate of mood and psychotic disorders

We first calculated the annual incidence per 100,000 individuals of newly diagnosed patients (both in- and outpatients) for each of the 27 municipalities within the department. For each year between 2005–2018, we divided the number of newly diagnosed patients in that specific year in each municipality by the estimated number of residents of that municipality in that year. The number of residents in a municipality was estimated using the WordPop database^22^, which includes gridded spatial demographic estimates. For most municipalities, the annual incidence of newly diagnosed cases was very low; we therefore conducted all our analyses based on the 14-year aggregate of the annual incidence. We subsequently referred to this aggregated value as the incidence rate and report this rate per 100,000 individuals.

We also calculated the incidence rate for each diagnosis, separately for in- and outpatients, at a more fine-grained geographic level unconstrained by municipality boundaries. To make these calculations, we imposed a grid (with a cell size of 5 km by 5 km) on the map of Caldas and counted the number of residents in each cell. Following the definition of the incidence rate at the level of the municipality, the incidence rate at the cellular level was defined as the number of newly diagnosed cases in the cell (between 2005 and 2018), divided by the total number of residents in that cell in the year 2018, and report this rate per 100,000. To visualize incidence rates, we generated maps using the R package ggplot2 v3.1.1 and shapefiles from the National Statistics Department of Colombia (DANE)^23^.

### Calculating a friction-surface, and a geographic accessibility, map

To quantify the geographic accessibility of mental healthcare for the population of Caldas, and to identify a relationship between the distribution of cases and the travel-time to the CSJDM, we constructed a friction surface map and subsequently a geographic accessibility map. We first plotted maps of Caldas using shapefiles from the National Statistics Department of Colombia (DANE)^23^. We then used the software package AccessMod5 v5.6.0^24^ to construct the friction surface map. This map quantifies the minimum amount of time that it takes to traverse one square kilometer, assuming driving time using a motorized vehicle on roads and walking time if no roads exist, while accounting for elevation changes and ground cover. To construct the map we combined geospatial data on topography^25^, land cover^26^, water bodies^27^ and road networks^23^ in the following stages: (1) We aggregated the topographic data to match the resolution (100 meters by 100 meters) of the WorldPop data. (2) We merged the land cover data, with data on roads and inland (permanent) water bodies, and with the aggregated topographic data. (3) We calculated travel-times using AccessMod, basing the estimated travel-time for each pixel on the quickest mode of transportation; Supplementary Table 1 lists the average speeds used to compute travel time, stratified by mode of transportation (see also Supplementary Material Section C in ref. ^28^). We then generated a geographic accessibility map by plotting the geographic location of the CSJDM on the friction surface map and computing the travel-time to the CSJDM from the centroid of each one square kilometer grid cell. We employed the option for anisotropic analysis, which uses the elevation data to modify travel speeds for walking.

### The incidence rate of mood and psychotic disorder in relation to the travel-time to the CSJDM

To make these calculations, we modeled the expected number of cases by travel-time in hours, accounting for the number of individuals in each grid cell of the geographic accessibility map. We used a zero-inflated negative binomial regression from the R package pscl v1.5.5, a procedure that accounts for both the large number of grid units with zero cases and over-dispersion of cases. Normal 95% confidence intervals and two-sided *p*-values were calculated for each relative risk (RR) based on the distribution generated from 1200 bootstraps using the R package boot v1.3-20. We performed this computation for inpatients and outpatients separately, considering: (i) all three diagnoses together (i.e., two comparisons), and (ii) the three diagnoses separately (i.e., six comparisons). We applied Bonferroni thresholds to correct for eight independent tests, with a significance threshold of 0.00625 (0.05/8). To evaluate possible heterogeneity in the observed relative risk by gender, we conducted sensitivity analyses, including in the model gender, and a gender by travel-time interaction.

### Identification of hotspots for residence of inpatients

To identify possible hotspots of residences of inpatients, we used the gridded map of Caldas showing the incidence rate of cases (for BPD, SCZ, and MDD combined, and separately). We then applied Kulldorff’s Spatial Scan Statistic^29^, implemented in the software package SaTScan v9.6^30^. SaTScan uses a circular window to scan a gridded surface. The window varies in size from one that encompasses a single grid cell to one that contains a predefined maximum population size. Use of a varying window size enables identification of hotspots unconstrained by municipality administrative boundaries. We used a cell size of 5 km by 5 km and set the maximum window size to encompass 25% of Caldas’ population. SaTScan identifies hotspots by maximizing a likelihood ratio function, and assesses their statistical significance using a one-sided Monte Carlo hypothesis procedure^29^. To account for multiple testing for four different tests, we applied a Bonferroni correction with a significance threshold of 0.0125 (0.05/4). As a sensitivity analysis, we also performed separate hotspot analyses based on gender. We selected 25% as the maximum hotspot size as it is frequently considered the largest window with potential future utility for policymaking^31^. To evaluate the effect of this threshold, we conducted a sensitivity analysis exploring, for each diagnosis, how the identified hotspots changed in size/location when varying the maximum allowable size of the reported cluster^32^.

We also employed SaTScan to quantify and visualize the uncertainty in defining the borders of hotspots, using Oliveira’s F function^33^. This function assigns an intensity value, ranging from zero to one, to each grid cell surrounding a hotspot, reflecting the probability that the grid cell belongs to the hotspot. To visualize hotspots and the uncertainty in their borders, we used QGIS v3.10^34^. The Oliveira’s F values were colored in categories split by Jenk’s natural breaks^35,36^, a setting in QGIS that enables better visualization of potential borders.

## Results

### Inequities in the geographic accessibility of mental healthcare in Caldas

**Fig. 1:**
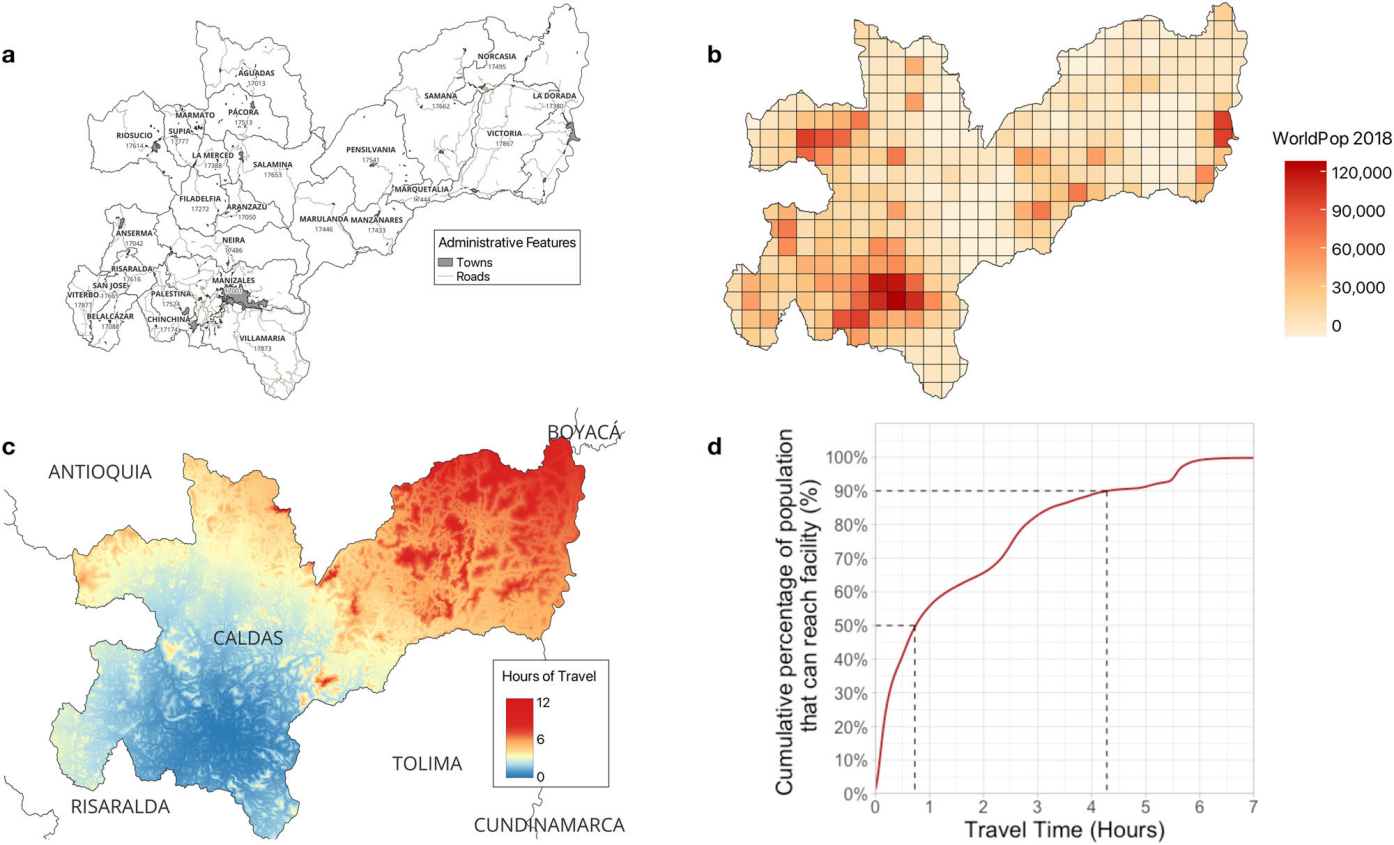
Caldas: administrative features, population density and access to mental healthcare. The maps show main roads and towns in Caldas^23^ (**a**), population size estimates for each 5 km x 5 km grid as specified in the WorldPop database^22^ (**b**), travel-time to Clínica San Juan de Dios Manizales (CSJDM, in hours) from anywhere in the department (**c**) and the percentage of the population of Caldas that can reach the CSJDM as a function of travel-time (**d**).

The Central Cordillera of the Andes Mountains divides Caldas into Eastern and Western sections, with the Western side containing the capital city, Manizales, and most towns (Fig. 1a, b). This division is evident in the geographic accessibility map (Fig. 1c), which shows the locations for which the CSJDM is the most inaccessible (travel-time (driving) of greater than 5 h, orange-red colored areas); virtually all these sites lie east of the Central Cordillera. While these relatively inaccessible locations, some of which are more than 10 h in travel-time from CSJDM, are geographically sizable, they include a relatively small proportion of the department’s population. Most of the population can access the hospital with less than 1 h of travel, while 90% can access it within approximately 4 h (Fig. 1d, see also Supplementary Table 2).

### Geographic variation in incidence rates

**Fig. 2:**
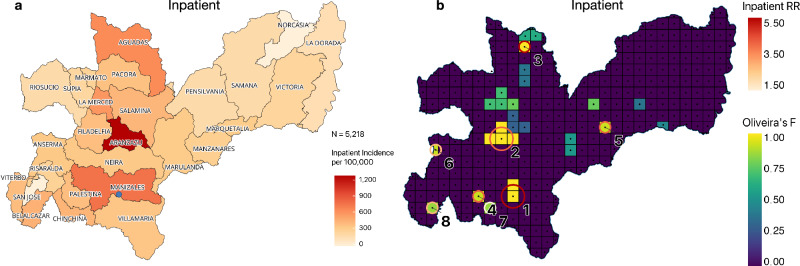
Observed patterns of the incidence rate for, and hotspots of, inpatients with mood and psychotic disorders. **a** The incidence rate for each municipality for patients whose first visit to the hospital was as an inpatient (per 100,000). The blue dot on the maps indicates the location of the Clínica San Juan de Dios Manizales (CSJDM). **b** Hotspots for all inpatients with bipolar disorder (BPD), major depressive disorder (MDD), or schizophrenia (SCZ). The statistically significant locations are indicated by circles: circle size indicates cluster size and color codes correspond to different values of relative risk. The grid units are colored by Oliveira’s *F* values.

**Fig. 3:**
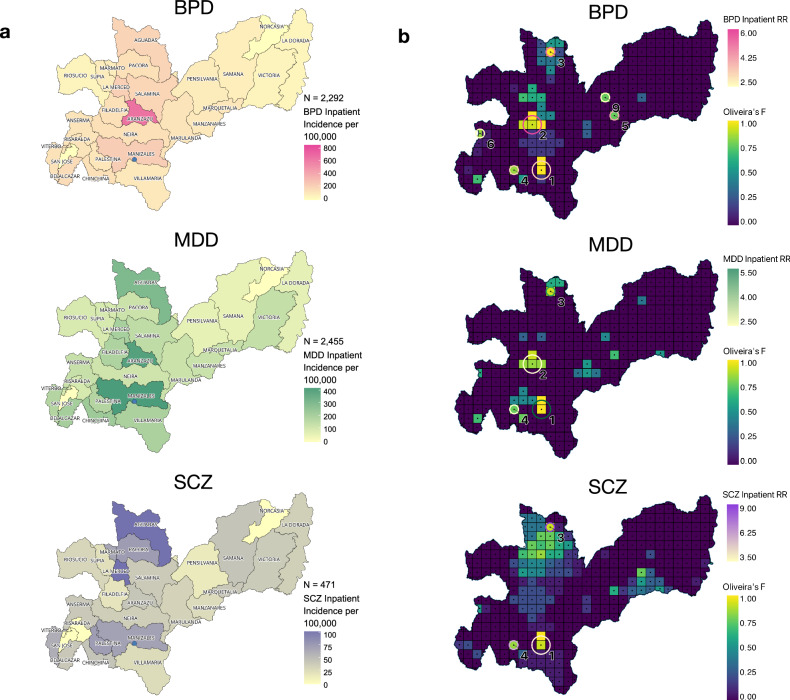
Diagnosis-specific observed patterns of the incidence rate for, and hotspots of, inpatients with mood and psychotic disorders. **a** The incidence rate for each municipality of inpatients stratified by diagnosis of bipolar disorder (BPD), major depressive disorder (MDD), and schizophrenia (SCZ) (per 100,000). The blue dot on the maps indicates the location of the Clínica San Juan de Dios Manizales (CSJDM). **b** Hotspots stratified by diagnosis. The statistically significant locations are indicated by circles: circle size indicates cluster size and color codes correspond to different values of relative risk. The grid units are colored by Oliveira’s F values.

The pattern of geographic variation in incidence rates, differs substantially between inpatients and outpatients (Figs. 2 and 3). For outpatients, by far the highest incidence rate is in the municipality (Manizales) which includes the CSJDM (Fig. 3). Notably, we found that the observed pattern for outpatients reflects strong distance-decay (as measured here by travel-times^8^); for every 1-h increase in travel-time to the CSJDM, the number of expected outpatients decreases by 20% (RR = 0.80, 95% confidence interval (0.71, 0.89), *p* = 5.67E-05, zero-inflated negative binomial model, Supplementary Table 3).

For inpatients, by contrast, we found no significant distance-decay, either for the incidence rate overall (i.e., for BPD, SCZ, and MDD combined) or for that of any of the three diagnoses considered separately (Supplementary Table 3. The overall incidence rate (Fig. 2a) is highest in a municipality, Aranzazu, located ~55 km from the CSJDM. This pattern reflects the high concentration of inpatient mood disorders (BPD and MDD) cases from this municipality compared to the department, overall (Fig. 3a): the incidence rate of inpatients with BPD is nearly fourfold higher (862/100,000 vs. 219/100,000), and the incidence rate of inpatients with MDD is more than 1.5 times higher (364/100,000 vs. 235/100,000). For MDD this incidence rate is nearly as high as that in Manizales (387/100,000). For SCZ (Fig. 3a) the incidence rate is highest in three neighboring municipalities located ~111 km from the hospital, where it is about twofold higher than in the department, overall (93/100,000 vs. 45/100,000). We found no significant interactions between travel-time and gender.

### Identification of hotspots for inpatients with mood and psychotic disorders

We identified nine distinct hotspots: eight hotspots were identified for the broad category of all inpatients with either BPD, SCZ, or MDD (Clusters 1–8, Table 2, and Figs. 2b, 3b), while one hotspot was unique to inpatients with BPD (Cluster 9). Seven of the nine hotspots were also found in our diagnosis-specific analyses (Clusters 1–6, 9, Table 2, and Figs 2b, 3b); i.e., they were hotspots for inpatients with either BPD, SCZ, or MDD.

**Table 2:** Hotspots for residences of inpatients.

Cluster	Latitude	Longitude	*N*	Population Size	Group	LLR	*P*-value	Observed Cases	Expected Cases	RR
**1**	5.04	−75.50	2	227752	Inpatients	1403.03	<1.00E-17*	2887	1128	4.49
					BPD	475.87	<1.00E-17	1169	496	3.78
					MDD	848.29	<1.00E-17	1476	531	5.47
					SCZ	100.44	<1.00E-17	242	102	3.84
**2**	5.27	−75.54	4	9118	Inpatients	107.47	<1.00E-17	174	45	3.95
					BPD	101.82	<1.00E-17	111	20	5.83
					MDD	20.75	9.19E-07	57	21	2.72
**3**	5.62	−75.45	1	3324	Inpatients	71.44	<1.00E-17	85	16	5.23
					BPD	27.59	1.59E-09	35	7	4.90
					MDD	28.77	8.04E-10	37	8	4.83
					SCZ	16.84	2.11E-05	13	1	8.98
**4**	5.04	−75.63	1	3409	Inpatients	31.87	1.45E-11	59	17	3.52
					BPD	12.86	8.57E-04	25	7	3.40
					MDD	10.53	7.22E-03	24	8	3.04
					SCZ	10.42	6.37E-03	10	2	6.69
**5**	5.31	−75.14	1	2069	Inpatients	22.12	1.24E-07	38	10	3.73
					BPD	12.91	8.25E-04	19	5	4.25
**6**	5.22	−75.80	1	5068	Inpatients	21.15	3.04E-07	64	25	2.57
					BPD	15.43	8.61E-05	34	11	3.11
**7**	5.00	−75.59	1	24106	Inpatients	18.15	4.88E-06	190	119	1.61
**8**	5.00	−75.81	1	3785	Inpatients	15.02	8.95E-05	47	19	2.52
**9**	5.40	−75.18	1	5337	BPD	11.14	4.03E-03	31	12	2.69

The rows describe the nine geographic locations with statistically significant overrepresentation of inpatient cases (Hotspots). Hotspots are numbered by the highest maximum LLR (log-likelihood ratio) in any of the four categories (inpatients overall, inpatients with BPD, SCZ, or MDD); all significant hotspots are listed. The latitude and longitude coordinates are listed for the center of each hotspot. “N” is the number of (5 km by 5 km) grid units within each cluster. “Cases Observed” is the number of cases in each hotspot, while “Cases Expected” is the number of cases expected according to a Poisson distribution in each hotspot. The cluster numbers correspond to those in Fig. 2.

*The lowest possible *P*-value obtained through this analysis is 1.00E-17, hence this is the lowest *P*-value reported.

In our diagnosis-specific analyses, we identified seven hotspots in total (Clusters 1–6 and Cluster 9, Table 2, Fig. 3b), all of which show a significant overrepresentation of inpatient BPD cases, and three of which (Clusters 1, 2, and 5) cross municipality boundaries. Cluster 2 is also a hotspot for MDD inpatients, while Clusters 1, 3, and 4 are hotspots for inpatients across all three diagnoses. The hotspot that includes the grid cells around the hospital (Cluster 1) displays the highest likelihood ratio for each of the three diagnoses, as well as for overall cases of MDD inpatients. This observation reflects the fact that many more patients reside in this metropolitan area than in any other region of the department; in particular, this hotspot is home to 1476 inpatients with MDD (60% of MDD inpatients in the department) and shows the greatest overrepresentation of this diagnosis (RR = 5.47, *p* = 1.00E-17). Other hotspots, however, show patterns of overrepresentation that appear to differentiate them by diagnosis. Cluster 9 (Fig. 3b) is specific to BPD and is not identified in the cross-diagnostic analyses.

Cluster 2 denotes the hotspot comprising the most extreme overrepresentation of inpatient BPD cases (RR = 5.83, *p* = 1.00E-17, Table 2 and Fig. 3b). It is the largest of the BPD hotspots in terms of geographical extent (covering four grid units: ~20 square kilometers) and crosses the administrative boundaries of two municipalities (Aranzazu, the municipality with the highest incidence rate of BPD [Fig. 3a/b] and Filadelfia); it is located about 1.5 h driving time from the hospital. This hotspot displays an extremely high average annual incidence of BPD hospitalizations (862/100,000) and also shows an overrepresentation of MDD but not of SCZ hospitalizations; it may therefore constitute a hotspot specific for inpatients with mood disorder diagnoses. Cluster 3, by contrast, appears to be a hotspot for inpatients overall, as evidenced by the overrepresentation of all three diagnoses, despite being located 5 h from the hospital. While the total number of cases of SCZ from this location is relatively small (*N* = 85 inpatients, N=13 with SCZ), the nearly nine-fold overrepresentation of SCZ cases is particularly striking (RR = 8.98, *p* = 2.11E-05, Table 2 and Fig. 3b).

Finally, we conducted a sensitivity analysis to explore the impact on the identified hotspots of varying the maximum allowable size of the reported cluster. The hotspots for MDD and SCZ are insensitive to the maximum scanning window size parameter as we observed nearly identical hotspots, irrespective of the parameters used (Supplementary Fig. 1). For BPD, when we increased the maximum reported cluster size above 40% of the population, Cluster 2 disappears. This is a consequence of the process by which SaTScan pre-computes the most likely hotspot centered at each grid, and then iteratively selects non-overlapping hotspots with the highest likelihood ratio; at settings above 40% the hotspot centered at Cluster 2 includes the area of Cluster 1. Since Cluster 1 has a higher likelihood ratio, Cluster 2 cannot be selected. The overall consistency of the hotspot patterns in these sensitivity analyses indicates the robustness of our findings. Our findings are also consistent when analyzed separately by gender; no new hotspots emerged, and we identify the same clusters described in Table 2.

## Discussion

Increasing the equitable availability and utilization of mental healthcare services is a major international development objective. Reducing inequities in the geographic accessibility of such services is critical to achieving this objective^37–42^ and, in turn, depends on detailed knowledge regarding variations in the geospatial distribution of mental health disorders and elucidation of the factors responsible for such variations. Within Latin America, geospatial methodology has been used to quantify geographic accessibility to different forms of healthcare^39,43^, but not to mental healthcare.

Our results show the inequitable geographic accessibility of specialty mental healthcare services in the department of Caldas, Colombia; while most of its population can access services at the CSJDM with a travel time of <1 h drive, a substantial proportion resides at >4 h drive distant from the facility. Our geospatial analyses of CSJDM EHR data reveal an important impact of this inequity, as utilization of outpatient care for mood and psychotic disorders demonstrates significant distance-decay in relation to the CSJDM. This observation aligns with findings from studies in UICs^1–5^, which have shown that most people will travel only short distances to obtain such care. The Government of Colombia recently enacted a strategy of increasing the geographical accessibility of mental healthcare by embedding it within primary care clinics distributed throughout the country^44,45^. Future geospatial investigations, including construction of geographic accessibility maps for primary care facilities and analyses of mental health treatment data from the EHRs of these facilities, could play a valuable role in evaluating the impact of this strategy.

Factors other than geographic accessibility, however, appear to be the main determinants of inpatient treatment for mood and psychotic disorders, which, as has also been found in UICs^46^, does not demonstrate significant distance-decay. Instead, utilization of such intensive treatment clusters significantly around nine hotspots, widely dispersed across the department of Caldas. This finding indicates that locally important sociodemographic, environmental, or genetic factors influence geospatial variation in risk, treatment-seeking behavior, or treatment accessibility for clinically severe presentations of these disorders.

Elucidating the above factors is a priority for future research aimed at reducing inequities in mental healthcare in this region, and an important early step will be to explain the approximately five-fold overrepresentation of BPD relative to SCZ in the CSJDM EHR database. Such an extreme excess of BPD among individuals hospitalized for mental disorders, has not, to our knowledge, been observed in studies of inpatients in other countries, where BPD and SCZ usually occur at roughly equal frequencies^47–49^. This excess is particularly striking in the observed BPD hotspots; for example, the hotspot at Cluster 2 displays an incidence for this disorder which may be the highest yet reported worldwide^48–50^. Systematic investigation of each of the hotspots, incorporating several additional types of data, may identify specific factors, contributing to these patterns.

Levels of socioeconomic inequality in Latin America, overall, are among the highest worldwide^51^, and have been associated with extreme disparities in utilization of mental health services^52^. Recent studies in Brazil and Peru have used geospatial analyses to document the relationship between inequalities in factors such as education levels, income, employment, and migration and geographic variation in the frequency of mental illness parameters (suicide and depressive symptomatology, respectively)^53,54^. By incorporating information on sociodemographic factors that is available at a detailed level throughout Colombia (e.g., each residence has an assigned socioeconomic status indicator, from 1 [lowest] to 6 [highest], which determines utility rates^39^), future studies, using sources such as Colombia’s National Department of Statistics census microdata^23^, could provide a more comprehensive equity perspective by integrating disaggregated georeferenced population sociodemographic open data.

Among the environmental factors that may contribute to geographic variation in risks of mental disorders, exposure to violent conflict, and forced displacement associated with such exposure are particularly relevant in Colombia; its decades long history of internal armed conflict led to the forced displacement of nearly 6 million people between the 1960s and the 2000s, one of the highest rates of any country worldwide^55,56^. Previous studies in Colombia have linked exposure to violent conflict to poor health outcomes generally^56^, and increased rates of mental health disorders, specifically^55,57^. Georeferenced resources, such as those aggregated by the government^58^, can be used to test this hypothesis.

An additional possibility, which can be investigated, is that genetic variants have contributed to the generation of hotspots for mood and psychotic disorders and to the overall patterns of geographic variation in their frequencies that we observe in Caldas. The demographic history of its population suggests this possibility. The predominant population of Caldas (“The Paisa”) is an example of a genetic isolate that has expanded rapidly subsequent to a series of migration-related bottlenecks^59,60^, from population founders of indigenous, African, and European ancestry^61^. Such rapid expansion from a bottleneck creates the opportunity for even deleterious variants to attain sizable frequencies – and thereby exert a substantial effect on disease risk – in a highly localized manner^62^. Because Caldas is part of a larger region in which the Paisa predominate (“The Paisa region”), extension of geospatial investigations to the EHR databases of healthcare facilities in neighboring departments provides an opportunity to determine the potential impact of such variants through well-powered genetic studies.

Finally, it is important to consider the possible impact of factors that may influence treatment-seeking behavior for mood and psychotic disorders but for which relevant data are currently unavailable and may be difficult to obtain on a sufficiently large scale to permit their use in detailed geospatial analyses. For example, several studies have suggested that stigma and the fear of discrimination may be important barriers to utilization of mental healthcare, in Colombia, but have focused on outpatient treatment^63^, excluded individuals living with psychotic disorders^17^ or were based on qualitative analyses of very small samples^64^.

We discuss here several limitations of this study. First, for simplicity sake our geospatial analyses have focused only on patients’ clinical presentations and diagnoses at their first visit, while a major strength of the EHR database is that it enables longitudinal investigations of variations in patient trajectories; many patients experience changes in the severity of their illness (e.g., their initial visit is as an outpatient, but they subsequently have at least one inpatient admission) or in diagnosis (e.g., they initially are diagnosed with MDD but subsequently experience manic or hypomanic episodes which leads to a change in diagnosis to BPD). Future analyses that incorporate information on such changes may reveal geographic variations that differ from those reported here. Second, as the size of the inpatient population at CSJDM is smaller than that of the outpatient population, it is possible that the lack of significant distance-decay in the former group reflects a lack of power to detect modest effects; future studies that include additional catchment areas may find some degree of distance-decay for these patients. Third, our estimates of geographic accessibility of treatment are based on average travel speeds using different surfaces, and do not account for potential seasonal or daytime variations. As more granular information becomes available (e.g., through dynamic road use databases), it may be possible to incorporate such variations as has been accomplished in Colombia for geospatial investigations of tertiary emergency healthcare^39^. Other directions for future work include the development of more sophisticated geostatistical models that could have policy relevance. For example, SaTScan maximizes the likelihood ratio and selects the most likely hotspot sequentially rather than jointly, which may not always lead to the selection of the most precise global pattern of hospots.

It is increasingly recognized that population-level investigations in low- and middle-income countries must be a priority for mental health research – both to reduce health inequities and to increase opportunities for transformative scientific discoveries^65^. The work reported here extends geospatial investigation of mood and psychotic disorders to a middle-income country that has recently experienced extensive and traumatic social disruptions. Despite such disruptions, Colombia has established infrastructure, such as the CSJDM EHR database, that constitute extraordinary resources for population mental health studies. Future studies can make further use of these resources to contribute to our understanding of sociodemographic, environmental and genetic risk factors of mental illness.

### Reporting summary

Further information on research design is available in the Nature Portfolio Reporting Summary linked to this article.

### Supplementary information

Supplemental Material

Supplementary Data 1

Reporting Summary

Description of Additional Supplementary Files

## Data Availability

Due to privacy protection concerns the local IRB committee prohibits the authors from making the dataset publicly available or available to other researchers. Source data are made available as Supplementary Data 1.
