# Supplementary material for: Geospatial investigations in Colombia reveal variations in the distribution of mood and psychotic disorders
Source: Commun Med (Lond). 2024 Feb 21;4:26. doi: 10.1038/s43856-024-00441-x (PMC10881503; doi:10.1038/s43856-024-00441-x)
Supplement: Supplementary file 1 — Supplemental Material [file 43856_2024_441_MOESM1_ESM.pdf]

Supplementary Materials to:

Geospatial investigations in Colombia reveal variations in the distribution of mood and psychotic disorders.

Janet Song; Mauricio Castaño Ramírez; Justin T. Okano; Susan K. Service; Juan de la Hoz; Ana M. Díaz-Zuluaga; Cristian Vargas Upegui; Cristian Gallago; Alejandro Arias; Alexandra Valderrama Sánchez; Terri Teshiba; Chiara Sabatti; Ruben C. Gur; Carrie E. Bearden; Javier I. Escobar; Victor I. Reus; Carlos Lopez Jaramillo; Nelson B. Freimer; Loes M. Olde Loohuis*; Sally Blower*

* shared last author

Supplemental items included:
Table S1: Travel speeds used to create a friction surface map of Caldas
Table S2: Proportion of the patient population that can travel to CSJDM within a specified travel time
Table S3: The effect of travel time on the incidence of severe and mild mental illness
Figure S1: Results of sensitivity analyses for hotspot identification

Class	Label	Speed	Mode
1006	caldas	50	MOTORIZED
1001	17	20	MOTORIZED
22	Mosaic_of_Cropland_Shrub_or_Herbaceous_cover	1.67	WALKING
19	Sparse_Herbaceous_or_sparse_Shrub_cover	2.5	WALKING
18	Urban_Areas	5	WALKING
17	Bare_Areas	2.5	WALKING
16	Mosaic_of_Cropland_Tree_cover_Other_Natural_Vegetation	1.67	WALKING
15	Cropland_upland_crops_or_inundated_flooded_crops_as_e_g_rice	1.67	WALKING
14	Regularly_flooded_2_month_Shrub_or_Herbaceous_cover_closed_to_open	1	WALKING
13	Herbaceous_cover_closed_to_open_15	1.67	WALKING
12	Shrubcover_closed_to_open_15_deciduous_broadleaved	1.67	WALKING
7	Tree_cover_closed_to_open_15_regularly_flooded_fresh_or_brackish_water_Swamp_Forests	1	WALKING
2	Tree_Cover_broadleaved_deciduous_closed_40	1	WALKING
1	Tree_cover_broadleaved_evergreen_closed_to_open_15	1	WALKING

Table S1. **AccessMod5 Travel speeds used to create friction surface map of Caldas.** Travel speeds used to generate the friction surface map stratified by different modes of transportation. Speeds are displayed in km/h.

Inpatient				Outpatient			
**Travel Time**	**Cat**	**count**	**Proportion**	**Travel Time**	**Cat**	**count**	**Proportion**
10	All	3171	0.61	10	All	7012	0.63
20	All	3267	0.63	20	All	7227	0.65
30	All	3360	0.64	30	All	7399	0.67
60	All	3737	0.72	60	All	8481	0.77
120	All	3955	0.76	120	All	8827	0.80
240	All	4743	0.91	240	All	10377	0.94
480	All	4940	0.95	480	All	10631	0.96
10	BPD	1272	0.55	10	BPD	1494	0.55
20	BPD	1317	0.57	20	BPD	1541	0.57
30	BPD	1357	0.59	30	BPD	1580	0.59
60	BPD	1536	0.67	60	BPD	1816	0.67
120	BPD	1672	0.73	120	BPD	1960	0.73
240	BPD	2065	0.90	240	BPD	2482	0.92
480	BPD	2155	0.94	480	BPD	2556	0.95
10	MDD	1644	0.67	10	MDD	5185	0.66
20	MDD	1688	0.69	20	MDD	5341	0.68
30	MDD	1733	0.71	30	MDD	5468	0.70
60	MDD	1892	0.77	60	MDD	6277	0.80
120	MDD	1966	0.80	120	MDD	6468	0.83
240	MDD	2270	0.92	240	MDD	7370	0.94
480	MDD	2339	0.95	480	MDD	7523	0.96
10	SCZ	255	0.54	10	SCZ	333	0.57
20	SCZ	262	0.56	20	SCZ	345	0.59
30	SCZ	270	0.57	30	SCZ	351	0.60
60	SCZ	309	0.66	60	SCZ	388	0.67
120	SCZ	317	0.67	120	SCZ	399	0.69
240	SCZ	408	0.87	240	SCZ	525	0.90
480	SCZ	446	0.95	480	SCZ	552	0.95

Table S2. **Proportion of the patient population that can travel to CSJDM within a specified travel time** Proportions stratified by inpatient/outpatient status and diagnosis. Travel time in minutes.

Encounter	RR	RR 95% confidence interval	RR p-value	OR	OR p-value
Inpatient	**0.88**	0.80 - 0.97	1.25E-02	0.33	1.10E-08
Outpatient	**0.80**	0.71 - 0.89	5.67E-05 *	0.28	1.51E-08
BPD Inpatient	**0.88**	0.79 - 0.99	3.61E-02	0.30	4.97E-08
BPD Outpatient	**0.84**	0.74 - 0.96	1.17E-02	0.29	2.01E-06
MDD Inpatient	**0.93**	0.84 - 1.03	1.76E-01	0.33	1.09E-08
MDD Outpatient	**0.79**	0.70 - 0.88	5.43E-05 *	0.31	4.27E-09
SCZ Inpatient	**1.05**	0.92 - 1.19	5.02E-01	0.23	1.60E-06
SCZ Outpatient	**1.00**	0.88 - 1.12	9.55E-01	0.37	1.25E-06

Table S3. **The effect of travel time on the incidence of severe and mild mental illness**
We employed a zero-inflated negative binomial model to assess the relationship between travel time and incidence of severe and mild mental illness in 372 grids of equal size in the department of Caldas. The results of the negative binomial count process are presented above as the Relative Risk (RR), RR 95% confidence interval, and RR p-values. We find the effect for each hour increase in travel time on the expected incidence overall and for individual diagnoses, in both cases considering inpatients (severe illness) and outpatients (mild illness) separately. P-values with an asterisk were significant after Bonferroni correction for analysis of eight tests P<0.0062 (0.05/8). The excess zero-process was modeled as a function of the log population size using logistic regression. The OR represents the change in odds of having a count of zero patients in a grid for each one-unit increase in log population size (a ~2.72x increase in population). BPD=bipolar disorder, MDD=major depressive disorder, SCZ=schizophrenia

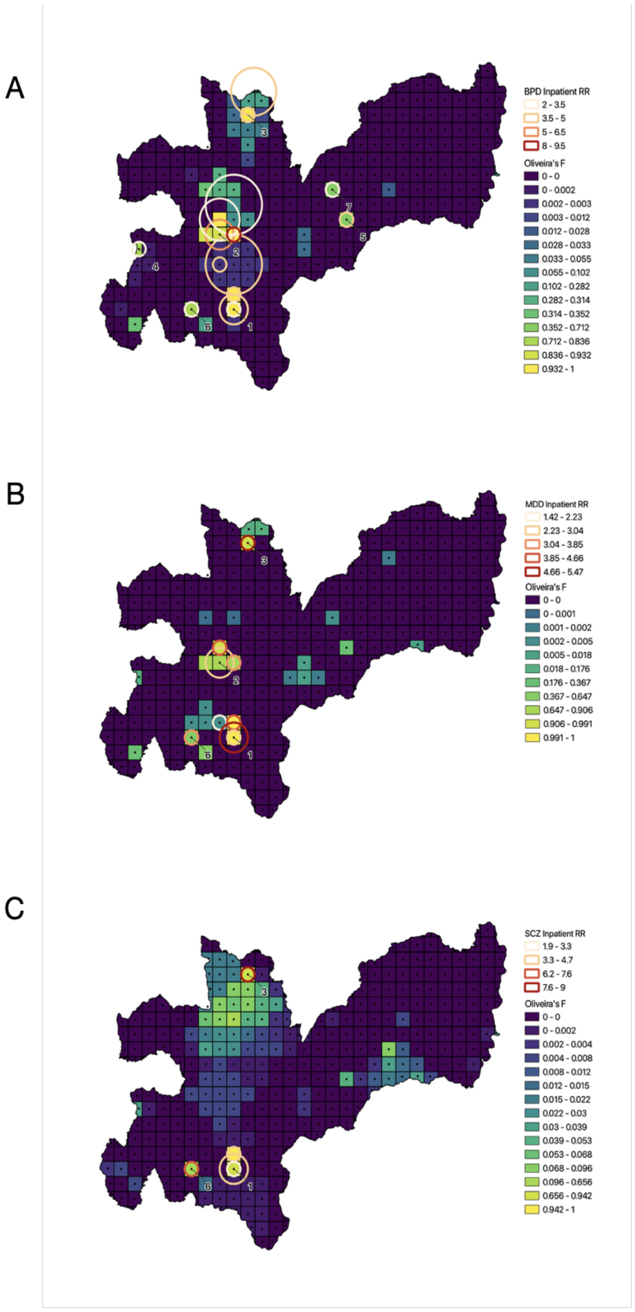

Figure S1. Results of sensitivity analyses for hotspot identification.
We analyzed the effect of varying the maximum reported cluster sizes in terms of the proportion of the total population of Caldas that they could include (5, 10, 15, 20, 25, 30, 35, 40, 45, and 50%). We conducted these analyses separately for BPD (A), MDD (B), and SCZ (C), displaying all hotspots that are significant for at least one of the above thresholds. The statistically significant locations are indicated by circles: circle size indicates cluster size and color codes correspond to different values of relative risk. The grid units are colored by Oliveira's F values at the standard threshold of 25% adopted in our primary analysis.
