# Supplementary figures and images for: Geospatial investigations in Colombia reveal variations in the distribution of mood and psychotic disorders
Source: Commun Med (Lond). 2024 Feb 21;4:26. doi: 10.1038/s43856-024-00441-x (PMC10881503; doi:10.1038/s43856-024-00441-x)

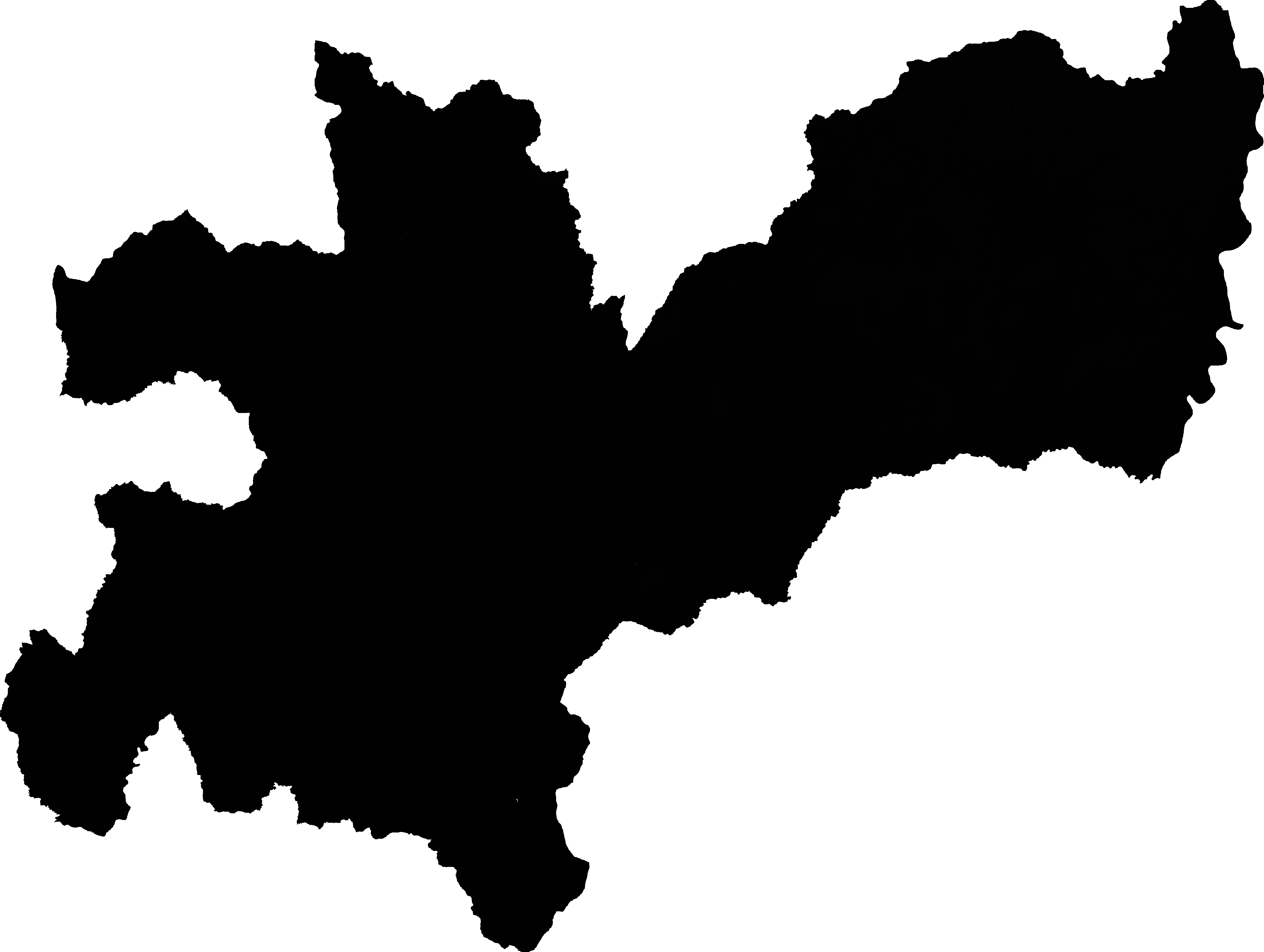

Supplement: Supplementary file 2 — Supplementary Data 1 [file 43856_2024_441_MOESM2_ESM.zip › Supplementary Data folders/Figure 1c/caldas_friction_surface_aligned.tif]
