# Supplementary material for: Geospatial investigations in Colombia reveal variations in the distribution of mood and psychotic disorders
Source: Commun Med (Lond). 2024 Feb 21;4:26. doi: 10.1038/s43856-024-00441-x (PMC10881503; doi:10.1038/s43856-024-00441-x)
Supplement: Supplementary file 3 — Reporting Summary [file 43856_2024_441_MOESM3_ESM.pdf]

nature portfolio

Corresponding author(s): Loes Olde Loohuis

Last updated by author(s): Apr 21, 2023

Reporting Summary

Nature Portfolio wishes to improve the reproducibility of the work that we publish. This form provides structure for consistency and transparency in reporting. For further information on Nature Portfolio policies, see our Editorial Policies and the Editorial Policy Checklist.

Statistics

For all statistical analyses, confirm that the following items are present in the figure legend, table legend, main text, or Methods section.

n/a	Confirmed	
☐	☒	The exact sample size (n) for each experimental group/condition, given as a discrete number and unit of measurement
☒	☐	A statement on whether measurements were taken from distinct samples or whether the same sample was measured repeatedly
☐	☒	The statistical test(s) used AND whether they are one- or two-sided<br>*Only common tests should be described solely by name; describe more complex techniques in the Methods section.*
☐	☒	A description of all covariates tested
☐	☒	A description of any assumptions or corrections, such as tests of normality and adjustment for multiple comparisons
☐	☒	A full description of the statistical parameters including central tendency (e.g. means) or other basic estimates (e.g. regression coefficient) AND variation (e.g. standard deviation) or associated estimates of uncertainty (e.g. confidence intervals)
☐	☒	For null hypothesis testing, the test statistic (e.g. F, t, r) with confidence intervals, effect sizes, degrees of freedom and P value noted<br>*Give P values as exact values whenever suitable.*
☒	☐	For Bayesian analysis, information on the choice of priors and Markov chain Monte Carlo settings
☒	☐	For hierarchical and complex designs, identification of the appropriate level for tests and full reporting of outcomes
☐	☒	Estimates of effect sizes (e.g. Cohen's d, Pearson's r), indicating how they were calculated

Our web collection on statistics for biologists contains articles on many of the points above.

Software and code

Policy information about availability of computer code

Data collection	Custom code was used to extract relevant features from the EHR database
Data analysis	Software used for analyses are (all are open access): OpenCage, R package ggplot2 v3.1.1, AccessMod5 v5.6.0, R package pscl v1.5.5, R package boot v1.3-20.SaTScan v9.6, QGIS v3.10. Confidence intervals are not reported by SatScan

For manuscripts utilizing custom algorithms or software that are central to the research but not yet described in published literature, software must be made available to editors and reviewers. We strongly encourage code deposition in a community repository (e.g. GitHub). See the Nature Portfolio guidelines for submitting code & software for further information.

Data

Policy information about availability of data

All manuscripts must include a data availability statement. This statement should provide the following information, where applicable:

- Accession codes, unique identifiers, or web links for publicly available datasets
- A description of any restrictions on data availability
- For clinical datasets or third party data, please ensure that the statement adheres to our policy

Data availability statement (to be included with the manuscript): Due to privacy protection concerns the local IRB committee prohibits making the dataset publicly available or available to other researchers. Source data are made available as Supplementary Data 1.

Human research participants

Policy information about studies involving human research participants and Sex and Gender in Research.

Reporting on sex and gender	Analyses were performed stratified by gender and aggregate statistics are included in the manuscript files.
Population characteristics	Population characteristics relevant and included in our analyses are gender, diagnoses, inpatient status and geolocated address
Recruitment	We used EHR records of all patients with Severe Menral Illness from a psychiatric clinic in Caldas Colombia
Ethics oversight	IRB committees from the clinic (Clinica San Juan de Dios), the University of Antioquia and UCLA approved the study

Note that full information on the approval of the study protocol must also be provided in the manuscript.

Field-specific reporting

Please select the one below that is the best fit for your research. If you are not sure, read the appropriate sections before making your selection.

☐ Life sciences ☒ Behavioural & social sciences ☐ Ecological, evolutionary & environmental sciences

For a reference copy of the document with all sections, see nature.com/documents/nr-reporting-summary-flat.pdf

Behavioural & social sciences study design

All studies must disclose on these points even when the disclosure is negative.

Study description	Quantitative EHR-based geospatial study
Research sample	We used EHR records of patients with Severe Mental Illness from a psychiatric clinic in Caldas, Colombia
Sampling strategy	na
Data collection	Prerecorded EHR data was extracted for the purpose of this study
Timing	Records from 2005 until 2018 were used for this study.
Data exclusions	Only participants without a geocoded address were excluded from analyses
Non-participation	na
Randomization	na

Reporting for specific materials, systems and methods

We require information from authors about some types of materials, experimental systems and methods used in many studies. Here, indicate whether each material, system or method listed is relevant to your study. If you are not sure if a list item applies to your research, read the appropriate section before selecting a response.

Materials & experimental systems

n/a	Involved in the study	
☒	☐	Antibodies
☒	☐	Eukaryotic cell lines
☒	☐	Palaeontology and archaeology
☒	☐	Animals and other organisms
☒	☐	Clinical data
☒	☐	Dual use research of concern

Methods

n/a	Involved in the study	
☒	☐	ChIP-seq
☒	☐	Flow cytometry
☒	☐	MRI-based neuroimaging
