# Supplementary material for: Geospatial investigations in Colombia reveal variations in the distribution of mood and psychotic disorders
Source: Commun Med (Lond). 2024 Feb 21;4:26. doi: 10.1038/s43856-024-00441-x (PMC10881503; doi:10.1038/s43856-024-00441-x)
Supplement: Supplementary file 4 — Description of Additional Supplementary Files [file 43856_2024_441_MOESM4_ESM.docx]

**Description of Additional Supplementary Files**

**File Name:** Supplementary Data 1

**Description:** Source data figures
